# Advanced Genetic Approaches in Discovery and Characterization of Genes Involved With Osteoporosis in Mouse and Human

**DOI:** 10.3389/fgene.2019.00288

**Published:** 2019-04-02

**Authors:** Jinbo Yuan, Jennifer Tickner, Benjamin H. Mullin, Jinmin Zhao, Zhiyu Zeng, Grant Morahan, Jiake Xu

**Affiliations:** ^1^School of Biomedical Sciences, The University of Western Australia, Perth, WA, Australia; ^2^Department of Endocrinology and Diabetes, Sir Charles Gairdner Hospital, Nedlands, WA, Australia; ^3^Research Centre for Regenerative Medicine, Guangxi Medical University, Nanning, China; ^4^The First Affiliated Hospital of Guangxi Medical University, Nanning, China; ^5^Centre for Diabetes Research, Harry Perkins Institute of Medical Research, The University of Western Australia, Perth, WA, Australia

**Keywords:** osteoporosis, GWLA, GWAS, PheWAS, WGS, collaborative cross, genome editing

## Abstract

Osteoporosis is a complex condition with contributions from, and interactions between, multiple genetic loci and environmental factors. This review summarizes key advances in the application of genetic approaches for the identification of osteoporosis susceptibility genes. Genome-wide linkage analysis (GWLA) is the classical approach for identification of genes that cause monogenic diseases; however, it has shown limited success for complex diseases like osteoporosis. In contrast, genome-wide association studies (GWAS) have successfully identified over 200 osteoporosis susceptibility loci with genome-wide significance, and have provided most of the candidate genes identified to date. Phenome-wide association studies (PheWAS) apply a phenotype-to-genotype approach which can be used to complement GWAS. PheWAS is capable of characterizing the association between osteoporosis and uncommon and rare genetic variants. Another alternative approach, whole genome sequencing (WGS), will enable the discovery of uncommon and rare genetic variants in osteoporosis. Meta-analysis with increasing statistical power can offer greater confidence in gene searching through the analysis of combined results across genetic studies. Recently, new approaches to gene discovery include animal phenotype based models such as the Collaborative Cross and ENU mutagenesis. Site-directed mutagenesis and genome editing tools such as CRISPR/Cas9, TALENs and ZNFs have been used in functional analysis of candidate genes *in vitro* and *in vivo*. These resources are revolutionizing the identification of osteoporosis susceptibility genes through the use of genetically defined inbred mouse libraries, which are screened for bone phenotypes that are then correlated with known genetic variation. Identification of osteoporosis-related susceptibility genes by genetic approaches enables further characterization of gene function in animal models, with the ultimate aim being the identification of novel therapeutic targets for osteoporosis.

## Introduction

Osteoporosis is a common skeletal condition that is characterized by low bone mass, abnormal mineralization, increased bone turnover rate and fragility (Burr, 2002). The diagnosis of osteoporosis relies on the measurement of bone mineral density (BMD) of the axial skeleton using dual energy x-ray absorptiometry (DXA) (Adler et al., 2000). Clinically, osteoporosis tends to remain undiagnosed until fracture has occurred (Unnanuntana et al., 2010). It is a highly prevalent condition affecting more women than men. Approximately 40% of Caucasian postmenopausal women are estimated to be affected by osteoporosis. Furthermore, the lifetime fracture risk for an osteoporotic individual is estimated to be as high as 40%, with fractures most commonly occurring in the spine, hip, or wrist (Burge et al., 2007; Unnanuntana et al., 2010).

**FIGURE 1:**
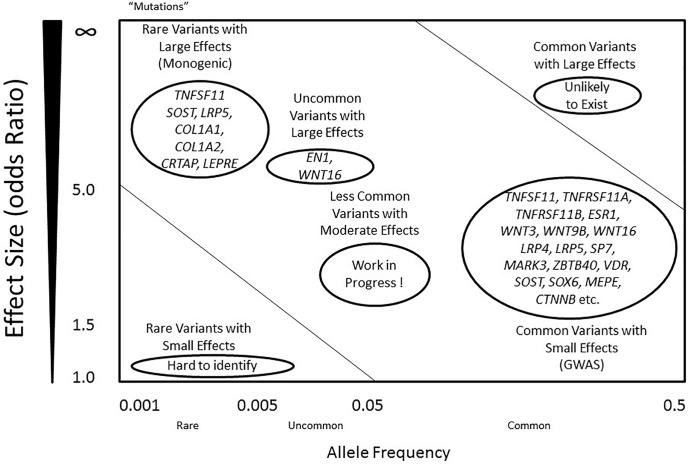
Genetic predisposition and architecture in osteoporosis. Allele frequency is defined as below: common (<0.5), uncommon (<0.05), and rare (<0.005). Mutations are considered as rare variants, mostly with an allele frequency less than 0.001, often with large effect sizes. Alleles that contribute to regulation of BMD include rare variants with large effects (left top), and common variants with small effects (right bottom). A few genes such as *COL1A1* and *LRP5* include variants that contribute to the phenotypes in either dominant or recessive mechanisms. However, most common variants within the genes present small effects, including *RANK*, *RANKL*, *OPG* and *LRP4*. Common variants with large effects are unlikely to exist, while rare variants with small effects are difficult to identify using current technology. Less common variants with moderate effects are likely to exist, and they may explain the majority of the missing heritability of osteoporosis.

The high risk of developing osteoporosis in specific populations suggests an underlying role of bone genetics in the development of this condition. Indeed, the genetic architecture of osteoporosis is typically complex, with contributions from multiple genetic loci and interactions with various environmental factors (Wade, 2001). With regards to the genetic aspect, even though more than 200 loci have been confidently linked to this complex condition, the genetic architecture, the number of susceptibility loci, the distribution of their allele frequencies, and the size of their effects, remains largely unknown (Ralston, 2010) (Figure 1). For most osteoporosis phenotypes, the majority of these genetic loci present small effects, given that few genes cause significant phenotypes in rare bone diseases, such as osteogenesis imperfecta and osteopetrosis, which follow Mendelian inheritance patterns and are caused by high penetrance mutations at a single locus (Rikhotso et al., 2008; Basel and Steiner, 2009).

Many osteoporosis-related traits such as BMD, bone loss, and osteoporotic fracture are highly heritable. BMD is considered to be a surrogate for osteoporosis and the strongest predictor of osteoporotic fractures (Black et al., 2018). A significant amount of the variance in peak BMD is genetically determined, with estimates ranging from 40 to 90% in various twin and family studies (Howard et al., 1998; Park et al., 2012; Hernandez-de Sosa et al., 2014). Twin studies have also revealed that genes account for 25–45% of the variation in age-related bone loss (Zhai et al., 2009; Mitchell and Yerges-Armstrong, 2011). Genetic factors also have a large contribution to fracture propensity. In the Framingham Heart Study (FHS) with a total of 4,134 cases of vertebral fracture, heritability was estimated to be up to 43% (Liu et al., 2012). The heritability of wrist and hip fractures was also estimated at 54 and 68% respectively, in peri-menopausal women (Andrew et al., 2005; Michaelsson et al., 2005).

Several methods have been widely used to identify osteoporosis-related genes. In this review, we will focus on the principles and the applications underlying these methods.

## Approaches to Identify the Genetic Architecture of Osteoporosis

### Genome-Wide Linkage Analysis (GWLA)

Genome-wide linkage analysis (GWLA) is a well-established genetic approach for identifying disease-risk genetic loci by scanning genome-wide markers in pedigrees segregating a trait. This approach can be categorized into two forms, parametric linkage analysis and model-free linkage analysis. The former is of most use in identifying mutations causing inherited monogenic Mendelian human diseases. The fundamental basis of this approach is looking for the segregation of gene variants (also called alleles) in diseases following dominant or recessive inheritance patterns within a family (Ralston, 2010). It has been employed successfully in identifying the causative gene for several monogenic diseases (Peltonen and McKusick, 2001). The latter form is used for complex traits with unknown inheritance pattern. In general, GWLA has shown less capability in identifying loci that are more likely to harbor genes for the complex traits or diseases as compared to Mendelian diseases/traits.

In bone, several attempts at using GWLA to detect loci that regulate BMD have failed to achieve consistent results and only very limited replication of linkage of peaks was found in different studies (Karasik et al., 2002; Shen et al., 2004; Ralston et al., 2005; Hsu et al., 2007). In efforts to improve the statistical power for detecting responsible loci, several meta-analyses of GWLA have been performed but have yielded limited success. For example, a meta-analysis containing 11 genome-wide linkage studies (total of 3,097 families with 12,685 individuals) for BMD or osteoporosis found seven linkage loci across studies but failed to provide evidence at the genome-wide significance level for any locus (Lee et al., 2006). Another similar large-scale meta-analysis with 11,842 subjects also failed to detect any genome-wide significant linkage associations, even though replication of several quantitative trait loci (QTL) from individual studies was identified (Ioannidis et al., 2007). This reflects the limited application of linkage analysis in the detection of genes modestly regulating BMD. GWLA remains as a powerful tool in identifying genes for Mendelian diseases/traits. However, during the past decade, this primary method for gene discovery has been replaced by another approach, the genome-wide association study (GWAS), which also exploits the linkage disequilibrium among dense markers in large populations.

### Candidate Gene Association Studies

Candidate gene studies were at the forefront of early efforts to identify osteoporosis susceptibility genes. The candidate gene approach focuses on assessing single nucleotide polymorphisms (SNPs) within genes that have a known role in a specific trait or disease. This approach has limited capability in gene discovery. Furthermore, it has been criticized on other aspects including lack of replication and lack of statistical power due to relatively small sample sizes (Wilkening et al., 2009; Patnala et al., 2013). Therefore, most of the results from candidate gene association studies should be interpreted with caution.

Approximately 200 candidate genes have been explored for their potential association with BMD or fracture in human populations (visit HuGE Navigator). A successful collaborative meta-analysis for osteoporosis candidate genes was performed using data from 19,195 subjects (including 14,277 women) from five Caucasian populations (Richards et al., 2009). Based on this study, researchers investigated 36,016 SNPs within 150 candidate gene loci and identified several SNPs from nine genes (*ESR1*, *LRP4*, *LRP5*, *ITGA1*, *SOST*, *SPP1*, *TNFRSF11A*, *TNFRSF11B*, and *TNFSF11*) as associated with BMD at either the femur neck or lumbar spine, with SNPs from four genes (*LRP5*, *SOST*, *SPP1*, and *TNFRSF11A*) significantly associated with fracture risk. The genetic effect sizes for BMD SNPs were also assessed, and results revealed no dominant allelic effects (Richards et al., 2009). Consequently, it was concluded that most genes selected for analysis in candidate gene studies for osteoporosis are likely not associated with bone phenotypes.

### Genome-Wide Association Study

**FIGURE 2:**
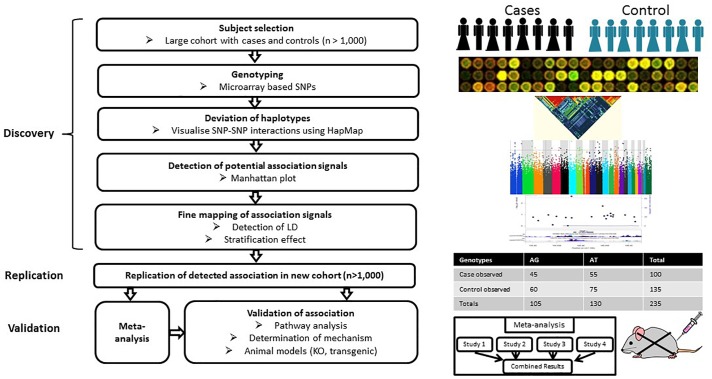
Workflow of GWAS. A typical GWAS includes three stages: discovery, replication and validation. The discovery stage focuses on identifying associations between SNPs and traits based on a large cohort with either quantitative trait or case/control phenotype data. The second stage focuses on the replication of preliminary associations in an independent cohort. Meta-analysis can be applied to increase the statistical power of individual GWAS at this stage. The final stage focuses on the validation of the detected associations through pathway analyses, determination of mechanism or genetic manipulation in animal models.

Genome-wide association study is hypothesis-free approach which appears to be more practical and effective than GWLA in the mapping of genetic loci associated with a complex disease or trait by scanning high-density markers distributed across the genome. It has greater statistical power than GWLA to identify common contributing genetic variants that do not follow Mendel’s laws of segregation (Hirschhorn and Daly, 2005). A typical GWAS includes several stages (Figure 2). The discovery stage focuses on identifying association between SNPs and traits in a large cohort with quantitative trait or case/control phenotype data. The second stage focuses on the replication of the most promising preliminary associations in an independent cohort. Meta-analysis of the discovery/replication cohorts can be applied to maximize statistical power at this point. The final stage focuses on the validation of the detected associations through pathway analyses, determination of mechanism or genetic manipulation in animal models. Currently, the genome-wide significance P-value threshold of 5 × 10^-8^ is generally applied to identify significant associations (Hoggart et al., 2008); however, other less stringent thresholds have also been employed (e.g., *P* ≤ 5 × 10^-7^) (Wellcome Trust Case Control Consortium, 2007).

The first recognized GWAS on bone phenotypes was published in 2007. In this study, 70,987 autosomal SNPs were evaluated in 1,141 patients (646 women and 465 men) against a range of phenotypes including BMD, broadband ultrasound attenuation (BUA), and fracture risk (Kiel et al., 2007). Even though several SNPs in genes such as *MTHFR*, *ESR1*, *LRP5*, *VDR*, and *COL1A1* showed evidence of association with osteoporosis, none of these variants achieved genome-wide significance due to the relatively small sample size and low genetic coverage. Subsequent studies published simultaneously in 2008 identified five loci that reached genome-wide significance with BMD, including *OPG*, *LRP5*, *ESR1*, *RANKL*, and *ZBTB40* (Richards et al., 2008; Styrkarsdottir et al., 2008). deCODE Genetics identified another four genome-wide significant loci near or at the genes including *SOST* (17q21), *MARK3* (14q32), *SP7* (12q13), and *TNFRSF11A* (18q21) (Styrkarsdottir et al., 2009).

Individual GWAS may be underpowered to detect all but the largest genetic effects on osteoporosis phenotypes because of the complex nature of the disease. Furthermore, the capture efficiency of GWAS varies due to genetic heterogeneity among study populations. Meta-analysis, a statistical procedure that integrates the results from multiple published or new studies, can be used in GWAS to provide a cost-effective way to increase statistical power, estimate the impact of gene-gene and gene-environment interactions and obtain *in silico* replication. For instance, a large-scale GWAS meta-analysis of five Northern European populations (total number = 19,195) conducted by the Genetic Factors of Osteoporosis (GEFOS) consortium, successfully confirmed seven genes as associated with osteoporosis (Rivadeneira et al., 2009). However, they found that the *MARK3* gene encoding microtubule affinity-regulating kinase 3, surprisingly, failed to achieve genome-wide significance. This study also identified 13 novel loci which containing 15 candidate osteporosis susceptibility genes reaching genome-wide significance across nine chromosomes (Table 1). In the larger GEFOS-2 analysis, with a total of 83,893 subjects from 17 GWAS and multi-ethnic groups, 56 loci including 32 novel loci reached genome-wide significance with either lumbar spine BMD, femur neck BMD, or both (Estrada et al., 2012). In addition, fourteen loci regulating BMD were also associated with osteoporotic fracture, of which six reached genome-wide significance. Interestingly, they could not find the significant association between SNPs in genes of the RANK-RANKL-OPG pathway and fracture risk, reflecting the heterogenous and complex nature of fracture risk.

**Table 1:** Genes with genome-wide significant evidence for association with BMD and osteoporosis.

Study	Type of study	Number of subjects	Key genes identified
Kiel et al., 2007	GWAS	1,141	“none”
Richards et al., 2008	GWAS	8,557	*TNFRSF11B (OPG)*, *LRP5*
Styrkarsdottir et al., 2008	GWAS	*13,786*	*OPG, TNFSF11 (RANKL), ESR1, ZBTB40*
Timpson et al., 2009	GWAS	*5,275 (children)*	*SP7*
Styrkarsdottir et al., 2009	GWAS	15,375	*SOST, MARK3, SP7, TNFRSF11A (RANK)*
Rivadeneira et al., 2009	GWAS; GWAS meta-analysis	19,195	*GPR177 (WLS), SPTBN1, CTNNB1, MEPE, MEF2C, STARD3NL, FLJ42280, LRP4, ARHGAP1, F2, DCDC5, SOX6, FOXL1, HDAC5, CRHR1, ZBTB40, ESR1, TNFRSF11B, LRP5, SP7, TNFSF11, TNFRSF11A*
Xiong D.H. et al., 2009	GWAS; GWAS meta-analysis	9,828	*ADAMTS18*, *TGFBR3*
Karasik et al., 2010	GWAS	2,073	“None”
Guo et al., 2010	GWAS	11,568	*ALDH7A1 (fracture)*
Kung et al., 2010	GWAS, GWAS meta-analysis	18,898	*JAG1*
Hsu et al., 2010	GWAS meta-analysis	11,290	*RAP1A, TBC1D8, OSBPL1A, GPR177, SOX6, TNFRSF11B*
Koller et al., 2010	GWAS	2,193	“None”
Duncan et al., 2011	GWAS	21,798	*GALNT3, RSPO3*
Estrada et al., 2012	GWAS meta-analysis	186,338	*RERE, ZBTB40, WLS, SPTBN1, GALNT3, CTNNB1, MEPE, MEF2C, CDKAL1*, *C6orf97, TXNDC3, STARD3NL, SLC25A13, C7orf58*, *WNT16, XKR9, TNFRSF11B, FUBP3*, *MPP7, MBL2, KCNMA1, SOX6, ARHGAP1, LRP5, SP7, HOXC6, AKAP11, RPS6KA5*, *AXIN1*, *SALL1, FOXL1*, *SMG6*, *C17orf53*, *SOX9, TNFRSF11A, GPATCH1, JAG1*
Zheng et al., 2012	GWAS; GWAS meta-analysis	5,672	*WNT16, TNFSF11*
Medina-Gomez et al., 2012	GWAS; GWAS meta-analysis	13,712	*WNT16*, *FAM3C*, *C7orf58 (CPED1)*
Paternoster et al., 2013	GWAS meta-analysis	10,452	*RANKL, LOC285735, OPG, ESR1, C6orf97, FMN2, GREM2*
Koller et al., 2013	GWAS meta-analysis	14,402	*WNT16, ESR1, C6orf97*
Zhang et al., 2014	GWAS meta-analysis	27,061	*SMOC1, CLDN14, ZBTB40, GPR177, FGFRL1, MEPE, MEF2C, C6orf97, ESR1, FLJ42280, SHFM1, FAM3C, WNT16, TNFRSF11B, SOX6, LRP5, AKAP11, FOXL1*
Moayyeri et al., 2014	GWAS meta-analysis	70,694	*TMEM135, ESR1, SPTBN1, RSPO3*, *WNT16, DKK1, GPATCH1*
Chesi et al., 2015	GWAS	1,399 (children)	*CPED1*, *WNT16*, *FAM3C, MIR31HG, MTAP*
Mullin et al., 2016	GWAS meta-analysis	6,696	*WLS*
Mullin et al., 2017	GWAS; GWAS meta-analysis	16,627	*PPP1R3B, LOC387810, SEPT5*
Kemp et al., 2017	*GWAS*	*142,487*	*ACTRT2, RERE*, *MTOR*, *SPEN*, *WNT4, ZBTB40, ARID1A, SF3A3, WLS, PKN2*, *WNT2B, TBX15, PRRX1, DNM3, HHAT, C1orf140, FMN2, TMEM18, PPP1CB, SLC8A1, C2orf91, THADA, SPTBN1, ZNF638, INSIG2, EN1, TANC1, FRZB, CDK15, FZD7, KIAA2012, ICA1L, NGEF, MLPH, ATG7, RARB, SUSD5, CTNNB1, ERC2, LEKR1, LINC00880, IDUA, FGFRL1, IGFBP7, SLC4A4, ARHGAP24, AFF1, DMP1, MEPE, SMARCAD1, OTUD4, ZNF827, PDGFC, CDH6, SLC1A3, DAB2, PLPP1, RASGRF2, CARMN, RREB1, CASC15, HLA-A, SUPT3H, GFRAL, BMP5, RSPO3, L3MBTL3, EYA4, ESR1, MEOX2, CREB5, AQP1, SFRP4, FAM133B, C7orf76, CPED1*, *WNT16, MPDZ, PAPPA, ABO, PLXDC2, MBL2, KCNMA1, SOX6, LRP4, LRP5, CADM1*, *WNT1, GPC6, BMP4, SMAD3, FTO, SMG6, SOST, TMEM92*, *AXIN2*, *TNFRSF11A, NFATC1, BMP2, JAG1, ERG, ITGB2, FAM9B*


*BMD loci that reached genome-wide significance (GWS; *P* < 5 × 10^-*8*^).*

In another GWAS meta-analysis published in 2014, 13 previously reported genome-wide significant loci were replicated in a cohort of seven GWAS samples consisting of 11,140 subjects (Zhang et al., 2014). In addition to replication of previously identified loci, two novel loci were identified, including 14q24.2 (rs227425, *SMOC1*) in the combined sample including both genders and 21q22.13 (rs170183, *CLDN14*) in the female-specific subset. These two SNPs collectively were found to explain 0.3–0.7% of phenotypic variation in BMD in this study. Combined with the other 13 SNPs, the cumulative effect of all the genome-wide significant SNPs on BMD in this study varied from 2.8 to 4.9% across population and skeletal site. As opposed to analyzing BMD and fracture risk at either the hip or spine using conventional DXA, an alternative approach is to focus on identifying genetic determinants of heel bone properties through quantitative ultrasound (QUS), which. This approach has the benefit of giving no radiation dose during measurement, being easily accessible, and demonstrates a high heritability of heel bone ultrasound values. In a GWAS meta-analysis published in 2014, nine SNPs had genome-wide significant associations with heel bone QUS, which included six previously reported genes (*ESR1, SPTBN1*, *RSPO3, WNT16*, *DKK1*, and *GPATCH1*) and two novel SNPs, including rs4869739 at the 6q25.1 (*ESR1*/*CCDC170*) locus, and rs597319 at the 11q14.2 (*TMEM135*) locus (Moayyeri et al., 2014). *TMEM135*, a gene recently associated with osteoblastogenesis and longevity, was significantly associated with both BUA and velocity of sound (*P* < 8.23 × 10^-14^). Another recently published meta-analysis of GWAS for QUS parameters of bone has identified an additional five novel loci for BUA, including at 8p23.1 (*PPP1R3B*, *FAM167A*, *DEFB103B*), 11q23.1 (*LOC387810*) and 22q11.21 (*SEPT5/TBX1*) (Mullin et al., 2017).

More recently, Kemp et al. (2017) performed a GWAS in 142,487 individuals (53% women) from the UK Biobank, and identified 203 loci (including 153 novel loci) achieving genome-wide significant associations with estimated BMD (eBMD) as determined using QUS measurements of the heel. Of these loci, 307 conditionally independent SNPs were identified, which were estimated to explain approximately 12% of the variance in eBMD. In particular, several variants (e.g., SNPs at *SEPT5*/*TBX1*, *ZNRF3*) presented extraordinarily strong association with heel eBMD (*P* < 10^-30^), which had failed to show any association in a previous study analyzing DXA-derived BMD (Zheng et al., 2015). These studies demonstrate the potential of QUS in the identification of osteoporosis susceptibility genes, with particular value in the analysis of extremities or in young subjects.

**FIGURE 3:**
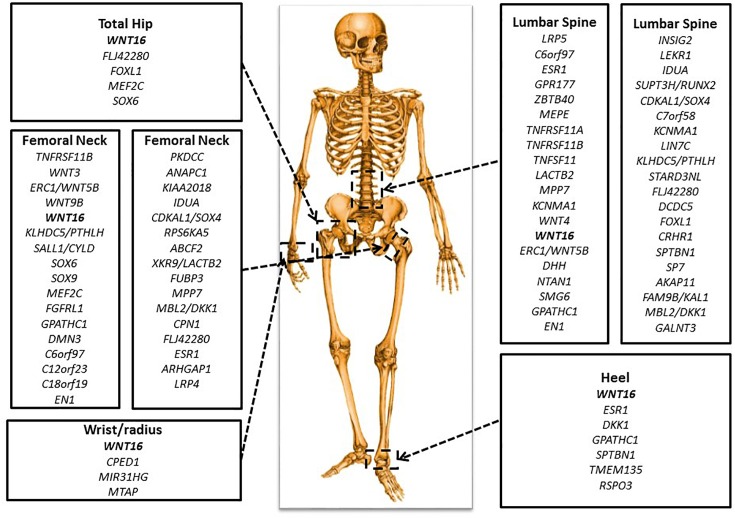
Important genetic loci associated with BMD. There are key genes identified in GWAS for BMD at various skeletal sites: total hip, femoral neck, lumbar spine, wrist or radius, and heel.

To date, over 220 genes within approximately 200 loci that have achieved genome-wide significance have been suggested based on these GWAS literature (see Figure 3 and Table 1). However, collectively, these genome-wide significant variants only explain approximately 20% of the genetic variance for this trait. Previously identified candidate genes such as *VDR*, *THFR* (methylenetetrahydrofolate reductase), and *IGF1*, have not been confirmed by any GWAS or GWAS meta-analysis (Rivadeneira et al., 2009; Estrada et al., 2012; Hsu and Kiel, 2012; Chesi et al., 2015). This phenomenon is not uncommon in GWAS due to the very strict genome-wide significance threshold resulting in possible false negatives. To interpret those inconsistent results between studies, as a rule of thumb, is suggested to consider a gene reaching genome-wide significance in any GWAS as “positive,” regardless of failure in achieving significance in other studies. Furthermore, statistical power can affect the outcome of GWAS. The larger the population size, the greater the power of the cohort to detect associations using the genome-wide threshold.

However, even if SNPs are identified as genome-wide significant, this phenotype-to-genotype approach often fails to establish clinically significant associations. Moreover, GWASs predominately identify common variants rather than uncommon or rare variants. Lastly, challenges exist in the characterization of the majority of GWAS SNPs which are intergenic. Establishing the function of these intergenic SNPs requires further or alternative strategies such as expression quantitative trait locus (eQTL) studies (Mullin et al., 2018, 2019), functional studies in animals, or the phenome-wide association study (PheWAS).

### Phenome-Wide Association Study (PheWAS)

**FIGURE 4:**
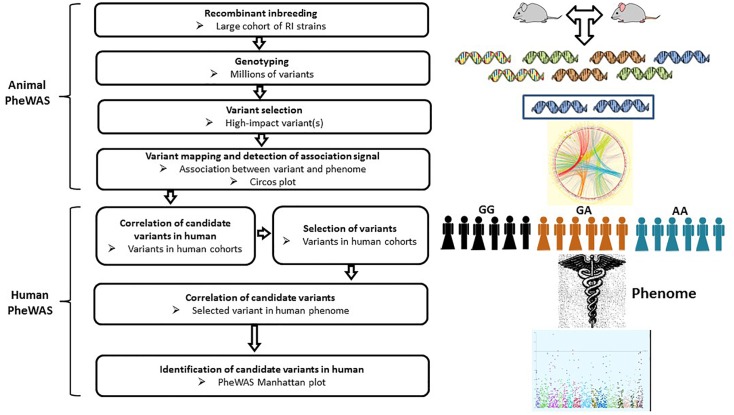
Workflow of PheWAS. The PheWAS can start with animal or directly with human cohorts. A typical approach is to identify high-impact variants within the RI strains, then correlate these variants with the mouse phenome, followed by validation of candidate variants in human cohorts.

PheWAS can be viewed as a reverse version of GWAS. Unlike GWAS, which applies a phenotype-to-genotype approach, PheWAS starts with a given single genetic variant to test for association with a wide range of human clinical phenotypes, the phenome (Figure 4). This genotype-to-phenotype strategy is a novel approach, with the first PheWAS as a proof-of-concept study published in 2010. This study demonstrated the feasibility of PheWAS to discover significant gene-disease associations (Denny et al., 2010). This technique is of particular usefulness in the re-discovery of known key genetic associations linked to immunological diseases (Hebbring, 2014). It also has the capacity to identify association between SNPs and bone phenotypes. For example, Wang et al. (2016) successfully identified a missense variant in collagen 6A5 (*Col6a5*) as associated with BMD, along with other numerous genotype-phenotype associations in a cohort of BXD recombinant inbred strains (*n* = 150), and a European human population (*n* = 29,722). PheWAS has a potential advantage over GWAS in being able to identify uncommon or rare variants responsible for bone phenotypes. However, this technique is significantly less efficient than GWAS in the discovery of genetic variants associated with bone diseases/conditions. Rather, PheWAS may be used as a complementary approach to either further investigate the associations between a genome-wide significant BMD-relevant SNP and clinical phenotypes beyond the skeletal system, or re-discover the association between a non-GWAS-significant BMD SNP and a wide spectrum of phenotypes including osteoporosis, osteopetrosis and osteoarthritis. Therefore, PheWAS has the potential to unlock novel genetic loci or variants that would not be possibly revealed by other genetic approaches.

### Whole Genome Sequencing (WGS)

Whole genome sequencing (WGS), also known as complete genome sequencing, is a technique that is capable of generating a complete set of DNA sequence variants rather than relying on the patterns of linkage disequilibrium for markers. Conventional sequencing of the whole genome of animal or human subjects is time-consuming; however, large scale WGS can be achieved using next-generation sequencing (NGS) platforms such as Ion Torrent’s PGM, Pacific Biosciences’ RS and the Illumina MiSeq (Quail et al., 2012). These sequencing techniques have been applied for complete characterization of candidate genes identified from GWAS.

Unlike GWAS, which relies on testing common variants (MAF ≥ 5%), WGS aims to assess the role of low frequency (MAF 1–5%) and rare (MAF ≤ 1%) genetic variation. A recent sequencing-based study, which included 4,931 Icelandic subjects with low BMD, along with population-based controls (total *n* = 69,034), identified a rare nonsense variant (c.376C > T) within the leucine-rich-repeat-containing G-protein-coupled receptor 4 (*LGR4*) locus as associated with low BMD and high fracture risks (Styrkarsdottir et al., 2013). Another study incorporating WGS, whole-exome sequencing and genotype imputation identified two novel low-frequency non-coding variants associated with BMD near *EN1* and *WNT16* in a total of 53,236 subjects (Zheng et al., 2015). The SNP (2q14.2, rs11692564[T], MAF = 1.7%) near *EN1* was found to have a larger effect size than previously reported common BMD variants for the lumbar spine, and was also associated with a low fracture risk (Estrada et al., 2012). Conditional knockout *En1^Cre/flox^* mice showed reduced bone mass, probably due to high bone turnover. Another SNP (rs148771817[T], MAF = 1.1%) near *WNT16* also showed significant association with BMD, adding evidence that this gene has large effects on osteoporosis across skeletal sites. Overall, these studies provide rationale for WGS to investigate the genetic composition of complex traits/disease in the global population. With dramatic reduction in the cost of WGS, this approach are expected to identify uncommon or rare variants which are likely missed by GWLA or GWAS. It could be applied for individual genome sequencing, which can guide the practice of personalized medicine.

### Animal Models

Animal models have been widely used to investigate the contribution of specific genes to the determination of bone mass and microarchitecture. In comparison with human populations, animal models offer several advantages, including a greater ability in environmental control, fast breeding rates, reproducible and easier access to trait-relevant tissues, the ability to easily monitor age-related phenotypes, and most importantly, genetic manipulations through gene knockout and knockin (allele swaps). Genetic manipulations through gene knockout and targeted mutations are of most importance for these animals models, and have been used to further evaluate the impact of loss-of-function or gain-of-function of osteoporosis susceptibility genes (e.g., *EN1* and *SOST*) identified from other genetic approaches or in human populations (Zheng et al., 2015; Sebastian and Loots, 2017).

Of those animals utilized in the past, mice and rats have been used far more widely than other animals such as rabbits, dogs, sheep or non-human primates (Jee and Yao, 2001; Shmookler Reis and Ebert, 2003). The mouse and human share high degree of similarity, with over 17,000 mouse protein coding genes have a known human homolog^1^. Such similarity provides a rationale that using mouse models to resemble genetic studies for humans, and mouse models have been considerably applied in studies of the genetic inheritance of bone mass, geometry and strength (Ishimori et al., 2006; Xiong Q. et al., 2009; Ackert-Bicknell et al., 2010; Jepsen et al., 2010; Smith et al., 2014). Hence for genetic analysis the mouse model is most efficacious, in particular for the study of common human skeletal diseases such as osteoporosis. However, we still need to be aware of that many findings in mice cannot resemble the gene-phenotype association in humans. Caution in translating such murine results in human populations is required.

### The Collaborative Cross (CC)

**FIGURE 5:**
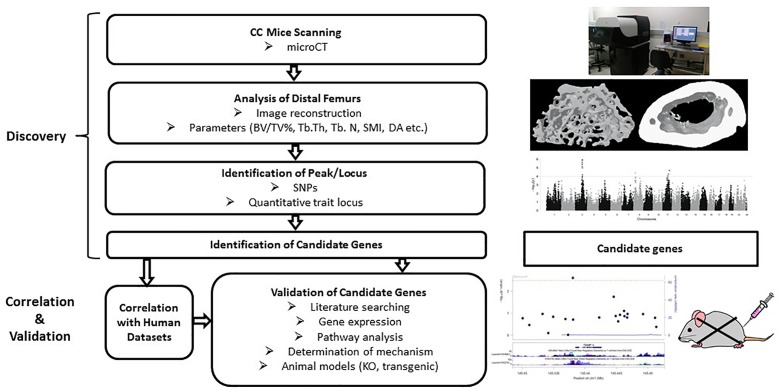
Workflow of the collaborative cross mice screening. The CC study starts with searching for associations among strains and identification of candidate genes within the identified locus, followed by validation of those candidate genes for association with the phenotype. Correlation with human datasets add confidence for the SNPs/genes. Validation can be done by gene expression, pathway analysis and looking at the functions of selected genes through transgenic or knockout mouse models.

The CC is essentially a form of GWAS in mice. It is a large-scale community-based project for developing recombinant inbred (RI) strains from eight fully genotyped parental strains that were carefully selected to catch nearly 90% of genetic variance in the mouse genome (Churchill et al., 2004; Morahan et al., 2008). The RI strains are generated by mating pairs of sibling F2 mice until fully inbred for each line, which requires 20 generations or more (Churchill et al., 2004). Because the CC strains are RI, genotyping of the mice are generally not required as their genotypes have been computationally resembled and are already available on public databases. The design of GWAS in CC mice fundamentally differs from the design of GWAS in humans (Figure 5). Analyzing traits of CC strains and searching for associations between phenotypes and genotypes are key steps in the study, followed by validation of genes of interest. The CC mice offer a novel avenue to explore the genetic architecture of complex traits such as osteoporosis.

Collaborative cross mice breeding is still in progress in order to achieve at least 1,000 independent lines and its application in genetic research is in its infancy. Generation of the targeted numbers of independent lines is unlikely to be achieved within the next decade due to limited ongoing funding and increased cost of animal handling and housing. Applications of CC mice over the past decade mainly focused on pre-CC mice, which are not fully inbred, and a wide range of phenotypes have been found in these studies (Bottomly et al., 2012; Phillippi et al., 2014; Ferguson et al., 2015). Inbred CC mice used in combination with other mouse models (e.g., transgenic) have also been applied, for example, in the identification of a mutation in the *Cdon* gene that accelerates congenital navus growth (Chitsazan et al., 2016). The function of this gene has now been further investigated using knockout mouse models (Chitsazan et al., 2017). Simulations of the power and precision of genetic mapping with the CC population demonstrated superior performance relative to traditional or alternative strategies (Broman, 2005; Valdar et al., 2006; Ram et al., 2014; Ram and Morahan, 2017).

So far, there is only one published study that analyzed genes for bone microarchitecture using a cohort of CC mice (Levy et al., 2015). This study utilized 160 mice across 31 lines to perform genome-wide haplotype mapping across 77,808 SNPs, and analyzed four trabecular traits (Levy et al., 2015). Haplotype association mapping revealed six QTLs, and numerous candidate genes such as *Oxt* and *Avp*. *Oxt* that encodes a precursor of oxytocin, and *Avp* encoding arginine-vasopressin appear to be promising since their proteins have shown a direct skeletal effect in other studies (Tamma et al., 2009; Sun et al., 2016). These findings provide a proof-of-concept that the CC is a powerful tool in the identification of genes that are responsible for complex traits or diseases. However, this study had several limitations such as small sample size (average of five mice per line), narrow age range (10–13 weeks), limited number of strains and limited parameters/traits. Such limitations may lead to a sub-optimal estimation of candidate genes responsible for bone microarchitecture. Further studies with a large scale of CC mice, therefore, are required.

### Mutagenesis and Characterization of Candidate Genes

Mutagenesis can be achieved either randomly or in a more controlled or direct way. Random mutagenesis is usually induced using chemicals such as *N*-ethyl-*N*-nitrosourea (ENU), which has proven to be an efficient way for generating mutations in phenotype-driven screening (Nelms and Goodnow, 2001; Stottmann and Beier, 2010). The high-throughput screening technique has previously unveiled numerous novel mutations that cause dysfunction of tissues and organs (Nelms and Goodnow, 2001). More recently, screening mice for mutations that affect bone phenotypes has been carried out. The screen involves assessing mouse bone density and skeletal structure in ENU-induced mutant mice (Mohan et al., 2007; Chen et al., 2014). This phenotype-driven approach enables a unique tool for studying genes regulating bone biology, including choline kinase beta (*Chkb*) (Kular et al., 2015), *Morc3* (Jadhav et al., 2016), and *Tnfsf11* (*Rankl*) (Douni et al., 2012).

Site-directed mutagenesis is another approach that utilizes plasmids within *Escherichia coli* (Shen, 2002). This approach was developed based on a battery of techniques including polymerase chain reaction (PCR), DpnI digestion, ligation and plasmid transformation (Shen, 2002). It usually yields mutations in over 50% of the desired mutated proteins, and allows study of the relationship between the gene sequence and its function. In the past, this technique was primarily targeted at one specific site using one set of primers. Recently, this technique has been modified, allowing site-directed mutagenesis at several sites within the plasmids without primers and ligation (Tian et al., 2010). Such advances significantly simplify the standard procedure in a more cost-effective way. So far, site-directed mutagenesis has been predominantly used in functional analysis of genes *in vitro*, including bone-related molecules such as bone sialoprotein (Harris et al., 2000), and neural epidermal growth factor-like (NEL)-like 1 (Takahashi et al., 2015). This technique provides an avenue to gene characterization *in vitro*, which is useful in linking a gene to a bone phenotype after gene screening by other approaches.

More recently, the field of genome editing has gained popularity and research interest. This technique encompasses a group of biotechnology tools (engineered sequence-specific nucleases) that allow researchers to modify the gene of interest. Those tools such as zinc-finger nucleases (ZNFs) (Carroll, 2011), transcription activator-like effector nucleases (TALENs) (Joung and Sander, 2013), and CRISPR/Cas9 (Ran et al., 2013), have been successfully applied to modify genomes with unprecedented efficiency and precision. ZNFs and TALENs can cleave sequences non-specifically, while the CRISPR/Cas9 system can cut the sequences more precisely using the guide DNA. With the commercial availability of thousands of customized guide DNA sequences, CRISPR/Cas9 is considered the most promising tool in gene editing among these nucleases (Williams and Warman, 2017; Cui et al., 2018). CRISPR/Cas9 has been used successfully to create animal models through microinjecting the customized guide DNAs that target bone genes such as osteocalcin (Lambert et al., 2016), and analyzing structural variations contributing to skeleton patterning in mice (Kraft et al., 2015; Lupianez et al., 2015). CRISPR/Cas9 may rapidly unveil the function of any newly identified osteoporosis susceptibility genes in bone and lead to effective treatment of this disease.

## Conclusion and Future Directions

Osteoporosis is a common but complex disorder, with only a few rare phenotypes inherited in a Mendelian pattern. Over the past two decades more than 220 genes have been associated with BMD or osteoporosis. However, less than 20% of BMD variation can be explained by those “confidently identified” genes. To identify the majority of the “missing” heritability requires further efforts in studies with larger populations across differing ethnicities, extensive application of current available genetic approaches, improvements in SNP coverage, and development of novel methods that can identify genotype-phenotype interaction in a fast, economic and precise way.

Each genetic approach has its strengths and limitations in discovering genes regulating bone mass (Table 2). Linkage analysis appears to have reached its limit in unveiling genes that can affect complex traits like BMD. Candidate gene association studies are useful in identification of the relationship between SNPs and traits within a suggested locus. GWAS, despite the huge breakthrough in its technique and application, only explains a small proportion of the total heritability of osteoporosis-related phenotypes, likely due in part to the large “false-negative” rate. It is realized that a GWAS provides more reliable results with a larger population size or combining published GWAS data *via* meta-analysis. In addition, it is worth mentioning that GWAS utilizing SNP array technology with subsequent genotype imputation tends to target common variants. As a novel genetic approach, PheWAS has been used as a complementary approach to GWAS in a reverse manner to identify variants associated with animal and/or human phenomes. It holds promise to discover uncommon or rare genetic variants which are usually missed by GWAS, despite that the application of PheWAS is still in its infancy. WGS also provides an alternative approach to exploring low-frequency variants or at all variants within a specific locus. With the dramatic reduction in cost, this approach allows the identification of more uncommon or rare variants and paves the way for personalized medicine. A combination of all these approaches with greater international collaboration allows researchers to identify more genes associated with complex diseases like osteoporosis. In addition, the majority of genes identified for BMD or osteoporosis are either rare mutations with large effects or common variants with small effects, focus should be shifted to searching for uncommon (1% ≤ MAF ≤ 5%) variants with modest effects. WGS appears to be more suitable than GWLA or GWAS to achieve this goal, if the cost of WGS becomes as low as one or two hundred United States dollars.

**Table 2:** Comparison of genetic approaches.

Approach	Strengths	Limitations
Genome-wide linkage analysis	✓ Systemically scan the genome ✓ Identify single gene with large effects	✓ Requires hundreds of family members ✓ Low resolution ✓ Limited power for complex traits/diseases
Candidate gene association studies	✓ Fine mapping ✓ Focused interest of gene	✓ Limited variants ✓ Not powerful in gene discovery
Genome-wide association study	✓ Hypothesis-free ✓ Systemically scan the genome ✓ Large variant coverage ✓ Detection of common alleles ✓ Powerful for complex traits/diseases	✓ Expensive ✓ Requires multiple tests ✓ High false-negative rate ✓ Usually fail to detect uncommon or rare variants
Phenome-wide association study	✓ Detection of uncommon or rare variants ✓ Re-discovery of known genes cross phenotypes ✓ Association of a single allele to phenotypes	✓ Definition of phenome is ambiguous ✓ Low phenotypic resolution
Whole genome sequencing	✓ Systemically scan the genome ✓ Detection of uncommon or rare variants ✓ Detection of variants in non-coding regions	✓ Expensive ✓ Platform dependant
Animal models	✓ Greater ability in environmental control ✓ Fast breeding rates ✓ Reproducible and easier access to trait-relevant tissues ✓ Short life span ✓ Genetic manipulations (gene-focused)	✓ Can resemble gene-phenotype effects in human
The Collaborative Cross	✓ Systemic scan within a defined RI family ✓ Availability of hundreds of strains ✓ Individual strains can be reproduced and used as mouse models	✓ Expensive in breeding and maintaining lines
Mutagenesis	✓ Create numerous mutants ✓ Rich variety of alleles	✓ Labor required ✓ May don’t get desired mutants


The CC and ENU mutagenesis mouse models offer another platform to identify genes responsible for osteoporosis and other complex diseases. The advantages provided by animal studies, including faster breeding, better control of environmental factors, and importantly, ease of genetic manipulation, make a strong case for their application in genetic research, although to date there are very few studies that have utilized CC mice and ENU mutagenesis. Genome editing tools, in particular CRISPR/Cas9, provide an effective way to dissect a gene that influences a specific trait or phenotype (e.g., BMD and osteoporosis), and direct therapies (e.g., gene therapy) to correct those defects induced by mutated or defective proteins. Given genome editing is in its infancy in guiding treatment of osteoporosis and other diseases, CRISPR/Cas9 appears to be a powerful tool in accelerating the CC mice screening process.

There is still much work to be done to fully characterize the genetic basis of osteoporosis. A greater understanding of the genetic variation underlying the development of peak bone mass, and rates of bone loss during aging, is required to develop strategies to optimize bone health in an aging population. Combining the available databases that link and characterize phenotypes provides us with an avenue to further investigate the mechanisms underlying the pathology. Moreover, osteoporosis is a complex condition which is influenced by a wide range of factors such as ethnicity, gender, age and environment. By far, most research focused on osteoporosis has been conducted in study populations of European origin. Further well-powered studies in other ethnic groups are necessary to provide a better picture of the correlation between the human genome and osteoporosis. With the knowledge gained from varied sources, personalized or precision treatment may be ultimately offered to patients based on the advances in understanding their “unique” genome through various genetic approaches, in addition to environmental factors.

## Data Availability

No datasets were generated for this study.

## Author Contributions

JY conducted the research and drafted the manuscript. JT, BM, and ZZ provided valuable opinions and assistance in the process of drafting and revision of the manuscript. JZ, GM, and JX supervised the study and revised the manuscript.

## Conflict of Interest Statement

The authors declare that the research was conducted in the absence of any commercial or financial relationships that could be construed as a potential conflict of interest.

## Footnotes

1 http://www.informatics.jax.org/homology.shtml
